# Relationship between chronic diseases and depression: the mediating effect of pain

**DOI:** 10.1186/s12888-021-03428-3

**Published:** 2021-09-06

**Authors:** Ying Ma, Qin Xiang, Chaoyang Yan, Hui Liao, Jing Wang

**Affiliations:** 1 Department of Health Management, School of Medicine and Health Management, Tongji Medical College, Huazhong University of Science and Technology, Wuhan, 430030 Hubei China; 2 The Key Research Institute of Humanities and Social Science of Hubei Province, Huazhong University of Science and Technology, Wuhan, 430030 Hubei China; 3 Institute for Poverty Reduction and Development, Huazhong University of Science and Technology, Wuhan, 430030 Hubei China

**Keywords:** Chronic disease, Depression, Pain severity, Pain relief

## Abstract

**Background:**

Chronic diseases have a high incidence in China and may cause pain and depression. However, the association of chronic diseases with pain and the incidence of depression has not been comprehensively investigated.

**Methods:**

The study population was obtained from the 2015 China Health and Retirement Longitudinal Study (CHARLS). The cross-sectional data from15,213 persons were included. CHARLS provides nationally representative data from21,097 individuals aged 45 years and older in approximately 150 districts and 450 villages. The main outcome was the incidence of depression. The main independent variable was chronic disease (no chronic disease, one chronic disease, and two or more chronic diseases). The mediators were the degree of pain (no pain, mild pain, and moderate to severe pain) and whether measures were taken to relieve pain (measures taken and no measures taken). We performed chi-square and binary logistic regression analyses of the associations of chronic disease with pain and the incidence of depression. The mediation model was examined using the Sobel test.

**Results:**

Patients with more chronic diseases had more severe pain (OR = 3.697, *P* < 0.001, CI = 2.919–4.681) and were more likely to develop depression (OR = 2.777, P < 0.001, CI = 2.497–3.090). The degree of pain partially mediated the interaction between chronic disease and depression in this study (t = 7.989, *P* < 0.001). The incidence of depression was high in people who were female, less educated, unmarried, living in rural areas, and working.

**Conclusions:**

The degree of pain had a partial mediating effect on chronic disease and depression. Pain relief measures should be considered when treating patients with depression.

## Background

The occurrence of chronic diseases is a predominant challenge in global public health [1, 2]. According to the 2019 study conducted by the Global Burden of Disease, chronic diseases, such as hypertension and diabetes, are included in the top five death risks in the world and are responsible for approximately 20 and 10% of global deaths, respectively [3]. According to the World Health Organization, 40.5 million (71%) of the 56.9 million global deaths in 2016 were due to noncommunicable diseases [4]. At present, the situation of chronic diseases in China is serious, and the prevalence of chronic diseases is increasing. In 2016, 230 million elderly people in China who were over the age of 60 years accounted for 16.7% of the total population, and the proportion of elderly people who were suffering from chronic diseases accounted for more than 65% of the elderly population [5]. In the USA, chronic diseases are the main cause of poor health, disability, and death and account for most healthcare expenditures. Approximately 60% of adults meet the criteria for multimorbidity in America, with a remarkable increase over the last three decades [6]. Half of the adults in Canada have at least one chronic disease, and a quarter have frequently occurring diseases [7]. These statistics show that chronic diseases have become the main cause of death and disease burden.

Patients with chronic diseases may experience negative emotions, such as depression. Many scholars have conducted related theoretical or empirical studies on the psychological status of patients with chronic diseases. The estimated prevalence of depression in patients with chronic diseases is ranges from 9.3 to 25% [8]. Patients with chronic diseases such as hypertension, coronary heart disease, and diabetes have a high incidence of depression; depression and the cardiovascular risk are strongly correlated [9]. People with multimorbid conditions are twice as likely to be depressed as people without multimorbidity [10]. Related studies have confirmed that many patients with chronic diseases have mental disorders, such as depression, because of the long-term nature of the disease and its impact on quality of life [11–13]. Patients with chronic diseases suffer from pain for many years and may even have physical dysfunction, which leads to a lower quality of life and lower ability for social and role adaptation than normal and subsequently leads to their denial of self-worth, a sense of powerlessness toward life, and the occurrence of depression [14].

In addition to depression, chronic diseases are also attributed to negative physical effects, such as pain. Chronic pain is the most common symptom of chronic diseases, such as rheumatic arthritis [15]. Chronic pain is experienced by 80% of patients with rheumatoid arthritis within 5 years of onset [16]. The most common types of pain in elderly adults are low back pain or neck pain, musculoskeletal pain, peripheral neuropathic pain, and chronic joint pain [17]. Approximately one- third of patients with chronic lung disease and coronary heart disease have chronic pain [18].

Patients who often suffer from pain are prone to depression and negative psychology. A 12-year longitudinal study on aging in Amsterdam concluded using covariate-adjusted Cox regression models concluded that the risk of developing depressive symptoms is substantial in nondepressed participants with elevated pain at baseline [19]. Depression and chronic pain are highly prevalent in elderly populations. Approximately 13% of the elderly population suffers simultaneously from pain and depression [20]. Clinical studies have revealed that chronic pain, a stress state, often induces depression, and up to 85% of patients with chronic pain are affected by severe depression [21–24]. Patients who are constantly in pain are more likely to develop muscle soreness, which may lead to irritability and restlessness and subsequently lead to anxiety or depression [25, 26]. Pain negatively affects the sleep duration, which also leads to an increased risk of depression [27]. People with chronic pain experience negative effects, such as loss of function, decreased quality of life, and depression [28]. The risk of depression increases remarkably as the pain level increases [29, 30].

Factors such as sex, age, and marital status may also have important effects on the relationship among chronic diseases, pain, and depression. Age, sex, marital status, and chronic diseases are related [31–35]. Sex is also associated with some chronic conditions [36]. Older age is a common factor associated with the four main chronic diseases, namely, hypertension, diabetes mellitus, chronic obstructive pulmonary disease, and stroke [36]. Age, sex, and marital status are associated with pain [37–41]. Women have lower pain thresholds and tolerance, and are more likely to experience more severe pain and unpleasant feelings than men [42–47]. Aging reduces pain sensitivity and pain intensity [37, 48]. Sex, age, and marital status are also associated with depressive symptoms [49–53].

Previous studies on chronic diseases have shown that chronic diseases directly lead to depression. Patients with chronic disease are affected by pain [15, 16, 18]. Patients who often suffer from pain have a higher incidence of depression than those who do not experience chronic pain [19]. Thus, studies are needed to verify whether pain exerts a mediating effect on the relationship between chronic disease and depression. This study used national survey data from middle-aged and elderly people to (1) explore the mediating effect of pain on the relationship between chronic disease and depression and (2) analyze the effects of demographic factors, such as sex, age, and marital status, on the relationships among chronic disease, pain, and depression.

## Methods

### Study setting

This study employed a cross-sectional design and was based on data from the 2015 China Health and Retirement Longitudinal Study (CHARLS). CHARLS is a nationally representative survey of individuals over age 45 in China. As of May 2017, three follow-up surveys had been conducted on sample populations in 150 counties and 450 communities or villages in 28 provinces (autonomous regions and municipalities) across the country in 2011, 2013, and 2015. In total, 12,241 households were selected for the survey, and 21,097 individuals were interviewed successfully.

All methods used in this study were implemented in accordance with relevant CHARLS guidelines and regulations. All participants joined CHARLS voluntarily and signed a consent form before participation. The original CHARLS was approved by the Ethical Review Committee at Peking University in June 2008 (IRB00001052–11015).

In the present study, participants aged over 45 years were selected from the 2015 CHARLS database. After the participants with missing key variables were removed, 15,213 participants, including 7484 males and 7729 females, were included in the analysis.

### Measurement

In this study, chronic disease was chosen as the independent variable. The chronic disease status of the respondents was divided into three categories: no chronic disease, one chronic disease, and two or more chronic diseases. The chronic diseases experienced by the respondents were categorized into 14 types, namely, hypertension, dyslipidemia (high cholesterol or low blood fat levels), diabetes or increased blood sugar levels, malignant tumor, chronic lung disease (such as chronic bronchitis and lung emphysema), liver disease (except fatty liver and liver cancer), heart disease (myocardial infarction, coronary heart disease, heart failure, etc.), stroke, kidney disease, gastrointestinal diseases, emotional or mental problems, memory- related diseases (Alzheimer’s disease, brain atrophy, Parkinson’s disease, etc.), arthritis or rheumatism, and asthma, to determine the number of chronic diseases.

The dependent variable was depression. This study used depression as a dichotomous variable based on the CHARLS survey questions about depression. The 10-item Center for Epidemiological Studies Depression Scale (CESD-10) in CHARLS is a simplified version of the depression scale developed by Radolff at the National Institute of Mental Health. The depression scores of each participant was evaluated using CESD-10. A score greater than or equal to 10 was considered to indicate depression, and a score below 10 was considered normal.

The mediating variables included pain severity and whether steps were taken to relieve pain. The degree of pain was set as the grade classification variable. In the CHARLS questionnaire, the question “On what part of your body do you feel pain? Please list all parts of the body where you are currently feeling pain.” requires individuals to enumerate pain sites. The number of pain sites was interpreted as follows: 0 as no pain, 1–3 as mild pain, and ≥ 4 as moderate to severe pain. According to the question “Are you taking measures to reduce the pain?”, the variable of taking steps to relieve pain was divided into yes (those who took Chinese traditional medicine, Western modern medicine, acupuncture treatment, professional massage therapy, or others) and no (those who did not take steps to relieve pain).

The covariates were the respondents’ sociodemographic characteristics, including sex, age (45–59, 60–69, and 70–101 years), educational attainment (uneducated, primary school, middle school, and high school and above), administrative registration status (rural and urban), marital status (unmarried and married), employment status, and insurance status. The detailed coding of each variable is shown in Table 1.

**Table 1:** Coding of variables

Variable	Coding
Chronic disease	No chronic disease = 0, One chronic disease = 1, Two or more chronic diseases = 2
Depression	No = 0, Yes = 1
Pain severity	No pain = 0, Mild pain = 1, Moderate to severe pain = 2
Take steps to relieve pain	No = 0, Yes = 1
Gender	Female = 0, Male = 1
Age	45–59 = 1, 60–69 = 2, 70–101 = 3
Education	Uneducated = 0, Primary school = 1, Middle school = 2, High school and above = 3
Urbanity	Rural = 0, Urban = 1
Marital status	Unmarried = 0, Married = 1
Employment status	No = 0, Yes = 1
Insurance situation	No = 0, Yes = 1

### Data analysis

First, the chi-square test was used to describe the differences in the incidence of depression among different attributes in the whole sample. Then, a binary logistic regression analysis was used to analyze the number of chronic diseases and the degree of pain (with mild pain as the reference), the relationship between the number of chronic diseases and whether measures were taken to reduce pain, and the influencing factors in individuals with self-reported pain. Next, the chi-square test was used to analyze the differences in the incidence of depression among patients with chronic diseases presenting different pain states. Binary logistic regression analyses and the Sobel test were used to explore the mediating effect of pain on the relationship between chronic disease and depression. Statistical analyses were performed using SPSS 12.0.

## Results

As shown in Table 2, among the 15,213 survey subjects, 18.16% had no chronic diseases, 26.60% had one chronic disease, and 55.24% had two or more chronic diseases. In addition, 33.90% had a tendency toward depression, 30.21% had moderate to severe pain, and 74.06% of the people with pain took measures to relieve pain. The incidence of depression was related to sex, age, education level, marital status, urbanity, current working status, chronic disease, and degree of pain in the comparison of the characteristics of the groups with and without depression. Participants who are female, middle-aged, less educated, unmarried, living in rural areas, working, suffering from a variety of chronic diseases, and suffering from severe pain had a higher incidence of depression. Insurance status and depression occurrence were not statistically significantly associated with depression. The participants with self-reported pain did not display substantial differences in the occurrence of depression, regardless of whether measures were taken to relieve pain.

**Table 2:** Sociodemographic data, chronic disease data, and the percentage of survey subjects reporting depression

Variable	Total	Depression		ϰ^2^	P
	(*n* = 15,213)	Yes	No		
**Sex (%)**				371.019	0.000
Male	49.19	26.43	73.57		
Female	50.81	41.22	58.78		
**Age (%)**				35.807	0.000
45–59	48.88	31.62	68.38		
60–69	34.19	36.47	63.53		
70–101	16.93	35.56	64.44		
**Education (%)**				543.129	0.000
Uneducated	40.91	43.27	56.73		
Primary school	24.34	33.23	66.77		
Middle school	22.04	26.63	73.37		
High school and above	12.61	17.87	82.13		
**Marital status (%)**				126.753	0.000
Married	87.55	32.31	67.69		
Unmarried	12.45	45.41	54.59		
**Urbanity (%)**				202.810	0.000
Urban	38.91	27.09	72.91		
Rural	61.09	38.31	61.69		
**Employment status (%)**				6.636	0.010
Yes	59.73	34.76	65.24		
No	40.27	32.74	67.26		
**Insurance situation (%)**				1.450	0.229
Yes	86.85	33.76	66.24		
No	13.15	35.13	64.87		
**Chronic disease (%)**				507.482	0.000
No chronic disease	18.16	19.8	80.2		
One chronic disease	26.6	28.29	71.71		
Two or more chronic diseases	55.24	41.31	58.69		
**Pain severity (%)**				2556.176	0.000
No pain	69.79	21.67	78.33		
Mild pain	11.77	50.06	49.94		
Moderate to severe pain	18.44	70.1	29.9		
**Take steps to relieve pain (%)**				0.001	0.974
Yes	74.06	62.28	37.72		
No	25.94	62.33	37.67		

Table 3 shows that in the patients with self-reported pain, chronic diseases and the degree of pain (with mild pain as the reference) were significantly related (odds ratio [OR] = 3.697, *P* < 0.001, confidence interval [CI] = 2.919–4.681). People with more chronic diseases had higher pain levels and were more likely to take measures to reduce pain. Sex, age, education, and urbanity were related to the degree of pain. Sex, age, and education were risk factors, whereas urbanity was a protective factor. Chronic diseases and pain measures were significantly positively correlated (OR = 1.693, *P* < 0.001, CI = 1.335–2.148). Sex was related to taking measures to reduce pain; namely, women were more likely to take measures to reduce pain than men.

**Table 3:** Binary regression analysis of the relationship between chronic diseases and pain in self-reported pain samples

Variable	Pain severity	Take steps to relieve pain
	OR (95% CI)	OR (95% CI)
**Sex (Male)**		
Female	1.499^***^ (1.314–1.710)	1.443^***^ (1.251–1.664)
**Age (70**–**101)**		
45–59	1.346^**^ (1.119–1.619)	0.958 (0.784–1.170)
60–69	1.135 (0.947–1.361)	1.042 (0.854–1.272)
**Education (High school and above)**		
Uneducated	1.647^***^ (1.273–2.130)	0.782 (0.584–1.047)
Primary school	1.298 (0.998–1.689)	0.745 (0.553–1.003)
Middle school	1.130 (0.861–1.484)	0.948 (0.694–1.295)
**Marital status (Married)**		
Unmarried	0.920 (0.769–1.100)	1.002 (0.824–1.219)
**Urbanity (Rural)**		
Urban	0.847^*^ (0.738–0.972)	0.921 (0.793–1.070)
**Employment status (Yes)**		
No	1.025 (0.897–1.172)	0.928 (0.803–1.196)
**Insurance situation (Yes)**		
No	1.067 (0.885–1.287)	0.978 (0.800–1.196)
**Chronic disease (No chronic disease)**		
One chronic disease	1.908^***^ (1.470–2.476)	1.246 (0.955–1.626)
Two or more chronic diseases	3.697^***^ (2.919–4.681)	1.693^***^ (1.335–2.148)

*P < 0.05, **P < 0.01, and ***P < 0.001, 95% CI = 95% confidence interval for the odds ratio

Table 4 illustrates that the number of chronic diseases and depression were substantially related to the pain level. Patients with more chronic diseases had a higher incidence of depression in the same pain state. Patients with more severe pain had a higher incidence of depression in the same chronic disease state. The number of chronic diseases was positively correlated with the incidence of depression regardless of whether measures were taken to relieve pain.

**Table 4:** Incidence of depression in individuals with different pain and chronic disease states

Variable	Depression incidence (%)			P
	No chronic disease	One chronic disease	Two or more chronic diseases	
**Pain severity**				
No pain	15.43	20.26	25.55	0.000
Mild pain	42.79	47.58	52.42	0.016
Moderate to severe pain	64.23	65.71	71.32	0.020
**Take steps to relieve pain**				
Yes	48.44	55.90	65.04	0.000
No	54.17	59.11	64.63	0.041

Table 5 displays the mediating effect of pain on the relationship between chronic diseases and depression, as determined using the binary regression analysis. In Model 1 shown in Table 5, sociodemographic characteristics, namely, sex, age, education, marital status, urbanity, current working status, and insurance situation, were controlled in the full sample, and the relationship between chronic diseases and depression was analyzed. Chronic diseases were significantly associated with depression (OR = 2.777, *P* < 0.001, CI = 2.497–3.090). The number of chronic diseases in patients was positively correlated with the incidence of depression. The incidence of depression was higher in patients with chronic diseases than in those without chronic diseases, and the incidence of depression in patients with more chronic diseases was higher than that in patients with one chronic disease. Sex, age, education, marital status, urbanity, and insurance status were related to depression. Among these variables, urbanity was a protective factor, and the remaining variables were risk factors.

**Table 5:** Binary regression analysis of the mediating effect of pain

Variable	Model 1 (*n* = 15,213)	Model 2 (*n* = 15,213)	Model 3^a^ (*n* = 4596)
	OR (95% CI)	OR (95% CI)	OR (95% CI)
**Sex (Male)**			
Female	1.666*** (1.546–1.795)	1.428*** (1.318–1.546)	1.300*** (1.138–1.486)
**Age (70–101)**			
45–59	1.189** (1.067–1.325)	1.107 (0.986–1.242)	1.011 (0.838–1.220)
60–69	1.144* (1.028–1.272)	1.100 (0.982–1.233)	1.057 (0.877–1.273)
**Education (High school and above)**			
Uneducated	2.456*** (2.139–2.819)	2.033*** (1.758–2.351)	1.621***(1.253–2.097)
Primary school	1.930*** (1.676–2.222)	1.641*** (1.415–1.904)	1.354* (1.040–1.763)
Middle school	1.530*** (1.325–1.766)	1.421*** (1.222–1.652)	1.265 (0.963–1.661)
**Marital status (Married)**			
Unmarried	1.496*** (1.344–1.665)	1.489*** (1.328–1.670)	1.426*** (1.182–1.722)
**Urbanity (Rural)**			
Urban	0.669*** (0.619–0.724)	0.734*** (0.675–0.798)	0.751*** (0.654–0.862)
**Employment status (Yes)**			
No	0.940 (0.870–1.016)	0.915* (0.842–0.994)	0.871* (0.761–0.997)
**Insurance situation (Yes)**			
No	1.178** (1.058–1.312)	1.141* (1.017–1.281)	1.120 (0.926–1.354)
**Chronic disease (No chronic disease)**			
One chronic disease	1.562*** (1.387–1.759)	1.332*** (1.177–1.508)	1.149 (0.887–1.488)
Two or more chronic diseases	2.777*** (2.497–3.090)	1.777*** (1.587–1.989)	1.478** (1.170–1.867)
**Pain severity (No pain)**			
Mild pain		3.124*** (2.809–3.474)	
Moderate to severe pain		6.421*** (5.834–7.078)	2.147 *** (1.889–2.440)
**Take steps to relieve pain (Yes)**			
No			1.149 (0.996–1.325)

*P < 0.05, **P < 0.01, and ***P < 0.001

^a^Models 1 and 2 use full samples. Model 3 uses pain samples

Model 2 further controlled the intermediary variable (pain severity) in the full sample. The degree of pain and depression occurrence were significantly correlated (OR = 6.421, *P* < 0.001, CI = 5.834–7.078). More severe pain was correlated with a greater likelihood of depression. In this model, the number of chronic diseases had less of an effect on depression. Sex, education, marital status, urbanity, work status, and insurance status were related to depression. The incidence of depression was higher in women, people with a low education level, unmarried people, people living in rural areas, working people, and uninsured people.

Model 3 used pain samples to further control whether measures were taken to reduce pain. No remarkable association between measures taken to reduce pain and depression occurrence was observed. Therefore, the effect of this mediating variable on depression should be verified. Chronic diseases and the degree of pain were still remarkably related to the occurrence of depression. Sex, education, marital status, urbanity, and employment were associated with depression. The incidence of depression was generally higher in patients who were female, less educated, unmarried, living in rural areas, and working.

According to these three models, patients with more chronic diseases were more likely to develop depression (OR = 2.777, *P* < 0.001, CI = 2.497–3.090), and the overall effect was significant. Patients with more chronic conditions had more severe pain (OR = 3.697, *P* < 0.001, CI = 2.919–4.681) and were more willing to take steps to reduce their pain (OR = 1.693, *P* < 0.001, CI = 1.335–2.148). Therefore, the independent variable (chronic disease) affected the two intermediate variables (pain severity and taking measures to reduce pain). Model 3 validates that taking measures to reduce pain and the incidence of depression were not considerably associated. Hence, the mediating effect of the degree of pain was considered. However, when the mediating variable (pain severity) was considered, the effect of chronic disease on depression decreased (OR = 1.777, *P* < 0.001, CI = 1.587–1.989). Therefore, mediating variables may produce partial mediating effects.

**Fig. 1:**
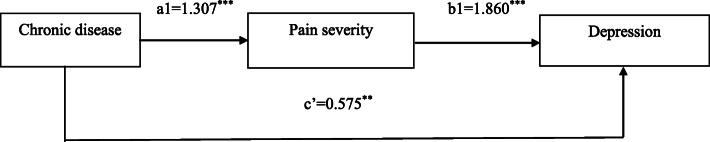
Final mediation model of mediation analysis

A Sobel test was carried out to further verify the mediating effect of pain severity (Fig. 1). According to the results, the mediating effect of pain severity was significant (t = 7.989, SE = 0.125, *P* < 0.001). The independent variable (chronic disease) affected the mediating variable (pain severity), and the mediating variable affected the dependent variable (depression). The direct effect (c’) of the independent variable (chronic disease) on the dependent variable (depression) was weakened when mediated by the mediating variable (pain severity). a1 = 1.307 and b1 = 1.860 for indirect effects, and a1b1 = 2.43 for the total effects.

## Discussion

The results of the study suggest that chronic diseases directly affect the occurrence and risk of depression through the mediating effect of pain severity. The degree of pain exerts a partial mediating effect on chronic diseases and depression. Patients with more chronic diseases had more severe pain and a greater risk of depression than patients with fewer chronic diseases. We used cross-sectional data in our research, but previous studies have shown that cross-sectional data may not be able to judge causality [54]. Therefore, chronic diseases may lead to depression, and depression may also lead to chronic diseases. Among the studies on the relationship between chronic diseases and depression, some studies have found that chronic diseases cause depression. For example, depression and the cardiovascular risk are strongly correlated, many patients with chronic diseases have mental disorders [8–10]. And some studies show that depression increases the risk of chronic diseases or aggravates the condition of patients with chronic diseases. For example, patients with major depression are at an increased risk for developing cardiovascular disease, depression mainly affects those with chronic medical illnesses, worsens the outcomes of many medical illnesses [55–57]. Although some studies may show that patients with depression are more likely to have chronic diseases, chronic diseases and pain caused depression in our study. Additionally, sex, education, marital status, place of residence, and work status were substantially associated with depression. Among people with chronic diseases and pain, people with the following characteristics were more likely to develop depression: women, people with lower education levels, unmarried people, people living in rural areas, and working people. We speculate that the direct effect of chronic diseases on depression may be due to physical dysfunction, social dysfunction, and the economic burden.

Many patients with chronic diseases have physical dysfunction, which adversely impacts daily life activities and leads to a decrease in quality of life. For example, patients with osteoporosis are prone to fracture and thus unable to carry out normal activities due to the gradual increase in bone fragility; hence, their daily activities are decreased [58]. Older people are prone to feelings of pain, anxiety, and even despair when they encounter difficulties in daily life [59]. Some patients with chronic diseases even must receive care from family members, which leads to an increased psychological burden and produces feelings of inferiority.

People with chronic diseases experience more negative life events than healthy people. Chronic diseases exert a negative effect on family relationships and social interactions and lead to more negative life events and stressful emotions [60]. One study found that negative life events are positively associated with depressive symptoms [61]. People with chronic diseases who experience more negative life events tend to have more severe depressive symptoms. According to stress theory, mental health tends to be poor when a person is stimulated by stress.

Another possible explanation is that patients with chronic diseases have a large amount of long-term medical expenses, which imposes a substantial burden on family and personal economies. Patients with chronic diseases who are unable to care for themselves require care from family members. Thus, the labor time of family members and the economic income of the family will be reduced, and the economic burden of the family will increase [62]. However, in Chinese tradition, most middle-aged and elderly people are expected to take care of their children or grandchildren. As a result, chronically ill elderly people who require care may see themselves as a burden on other family members, which may lead to feelings of despair [63].

The mediating effect of pain on the relationship between chronic disease and depression may be because pain is one of the symptoms of chronic disease. Chronic diseases, such as arthritis, rheumatism, and gastrointestinal diseases, are often accompanied by pain symptoms [64]. Chronic diseases are long-course diseases. Therefore, the pain caused by chronic diseases is also long term. Pain caused by physical discomfort results in a decrease in the quality of life of patients and results in irritability, restlessness, and other negative emotions, which all lead to the occurrence of depression [28]. Patients with multiple chronic diseases may experience a greater degree of pain. Patients may feel uneasy, powerless, irritable, and other negative emotions after the long-term accumulation of pain, which results in a substantial increase in the risk of depression.

Among people with chronic diseases and pain, individuals with the following characteristics are more likely to become depressed: women, people with lower education levels, unmarried people, people living in rural areas, and working people. The potential explanations are described below. Women with chronic diseases and pain are more likely to be depressed, possibly because women have a lower tolerance for pain than men. A study on pain conducted in Maine, USA, found that women have a lower pain threshold and tolerance and are more likely to experience more intense pain and unpleasant feelings than men [46, 47]. In addition, people of different sexes experience pain differently because of hormonal and genetic factors [39]. Older women with lower education levels and older women in rural areas usually have a lower socioeconomic status and receive less social support, which increases the risk factors for depression [65, 66]. Moreover, residents of rural areas have limited living environments and material resources, and thus they are unable to obtain complete treatment when suffering from chronic diseases and pain. Therefore, they suffer physically and psychologically [40]. On the one hand, unmarried people may lack the supervision of a partner and may have lower adherence to medication for a chronic illness. On the other hand, unmarried people lack the mental support from their partners and are more prone to depression when suffering from chronic diseases and pain [67, 68]. People with jobs are more likely to suffer from depression than those without jobs. One of the reasons is that people with jobs may suffer from an irregular diet due to busy work and become prone to chronic diseases, such as chronic gastritis and shoulder and neck pain [41]. Another possible explanation is that people with jobs may suffer pressure from work, family, and society, which may increase the psychological burden. Working people are more prone to depression due to the dual effects of physical discomfort and psychological stress.

The current study has some limitations. First, pain and depression were not analyzed in participants with different chronic diseases because of data limitations. Detailed information on the severity of chronic diseases was not included. Only the associations of the number of chronic diseases and pain with depression were emphasized. Therefore, the different effects of different chronic diseases on depression may be overlooked. Further studies on individuals with different chronic diseases should be conducted in the future. Second, chronic diseases were not verified from medical records; therefore, the study may be subject to recall bias. Patients may conceal diseases or report diseases that have been self-diagnosed but not recorded by medical institutions, leading to underreporting or overreporting. Third, we used cross-sectional data, which are unable to predict a causal relationship between chronic diseases and depression. Evidence suggests that cross-sectional approaches to mediation generate substantially biased estimates of longitudinal parameters in the special case of complete mediation [54, 69]. The results of our study may also suggest that individuals who are depressed are more likely to experience chronic diseases and pain. However, in our study, pain was added as a mediating variable between chronic diseases and depression to explore their relationship. Hence, pain is not necessarily related to time. The pain caused by chronic diseases may be temporary and could be relieved in a timely manner through drug intervention. Therefore, the use of cross-sectional data might achieve our research goals. In future studies, we will consider the use of longitudinal data for the mediation analysis.

## Conclusions

The effect of chronic diseases on the psychological status of middle-aged and elderly people can be partially predicted by determining their pain severity. This study emphasizes that depression might be avoided in some patients with chronic diseases by relieving pain, ameliorating the disability and economic burden caused by chronic diseases. Therefore, measures that reduce pain should be considered in the future treatment of patients with both chronic diseases and depression.

## Data Availability

The datasets analysed during the current study are available on http://charls.pku.edu.cn/pages/data/2015-charls-wave4/en.html.

## Abbreviation

CHARLS: China Health and Retirement Longitudinal Study
